# Editorial: Anesthesia and cancer: Friend or foe?

**DOI:** 10.3389/fonc.2022.1095800

**Published:** 2022-12-19

**Authors:** Lucillia Bezu, Oscar Díaz-Cambronero, Oliver Kepp

**Affiliations:** ^1^ Equipe Labellisée Par La Ligue Contre Le Cancer, Université de Paris, Sorbonne Université, INSERM UMR1138, Centre de Recherche des Cordeliers, Institut Universitaire de France, Paris, France; ^2^ Metabolomics and Cell Biology Platforms, Gustave Roussy Cancer Campus, Université Paris Saclay, Villejuif, France; ^3^ Gustave Roussy, Département d’Anesthésie, Chirurgie et Interventionnel, Villejuif, France; ^4^ Euro-Periscope: The Onco-Anaesthesiology Research Group (RG) of European Society of Anaesthesiology & Intensive Care (ESA-IC), Brussels, Belgium; ^5^ Hospital Universitario y Politécnico la Fe, Department of Anaesthesiology, Valencia, Spain; ^6^ Perioperative Medicine Research Group, Instituto de Investigación Sanitaria la Fe, Valencia, Spain

**Keywords:** anesthesia, cancer, perioperative care, immunity, surgery

Until now, the main objective of anesthesia has been efficient hypnosis and analgesia that are compatible with organismal homeostasis and the subsequent recovery of patients undergoing surgery. Over the past decade, some preclinical and observational clinical studies pointed towards the fact that certain anesthetic agents can impact therapeutic effects in cancer patients, for instance by modulating the rate of recurrence after oncological procedures (1–3). However, depending on the type of anesthetic and the clinical protocols employed, both pro- or antitumoral effects have been reported and therapeutic consequences have been debated (4). Thus, direct cytotoxic effects of local and systemic anesthetic agents such as lidocaine, ropivacaine, and propofol have been described suggesting anticancer effects (5, 6). However, morphine reportedly activates matrix metalloproteinases that then would promote the dissemination of tumors (7). Moreover, perioperative immunomodulatory factors such as undernutrition, anemia, neoadjuvant chemotherapy, as well as the concomitant use of mechanistically distinct anesthetic agents during oncosurgery, render the translation of partially promising preclinical results into clinical practice difficult. Altogether, results from preclinical studies stay conflicting, and clinical data are limited to retrospective studies that often are biased by confounding factors. Nevertheless, the potential impact on oncological patient care warrants further research for establishing guidelines on the use and regimens of anesthetic agents in oncosurgery.

The present Research Topic summarizes available data on potential anticancer effects of currently employed local, regional and general anesthesia that have been described in preclinical studies, as well as in prospective and retrospective clinical trials.

Several articles described the impact of anesthetic agents on the metabolism and survival of malignant cells, as well as on cancer immunity in various types of cancer cells. In their review, Chuang et al. summarize the direct cytotoxic effects and indirect immune-mediated antitumor properties of local anesthetics used as standalone agents or combined with conventional antineoplastic therapies. Bezu et al. describe the epigenetic changes induced by local anesthetics, which can impact on tumor cell survival, proliferation and migration by an increase in the expression of tumor suppressor genes. In an original study, Shin et al. describe the effect of dexmedetomidine on the proliferation of SK-OV-3 ovarian cancer cells and on the Natural Killer cell activity in a tumor xenograft established in mice. Belltall et al. describe a potential association between the variation of opioid receptor gene expression and the incidence of neoplastic recurrence using a compendium of preclinical and clinical research methods. Fang et al. review the mechanisms of propofol and sevoflurane on cellular proliferation, migration, cell death and anticancer immunity in breast cancer. Finally, Huang et al. show *in vitro* anti-breast cancer effects induced by tramadol, an antalgic opioid currently used to minimize postoperative pain. Cell growth, invasion, migration and metabolism were monitored after exposure to tramadol alone, and synergistic effects were described for tramadol co-administered with doxorubicin.

Another set of articles explored the indirect effect of pain control and immunomodulation on cancer prognosis. Moorthy et al. furnish a systematic review addressing the question as to whether anesthesia techniques and analgesia management optimizing acute pain can control the risk of relapse and dissemination. In yet another review, Zhang et al. discuss the use of intra- and postoperative epidural anesthesia for reducing the consumption of potentially pro-tumoral opioids and volatiles. Moreover, they analyze the capacity of epidural anesthesia to indirectly control inflammatory responses.

Further articles reveal novel mechanisms induced by anesthetic agents that can control malignant progression. Thus, Zhu et al. propose an inhibitory effect of certain anesthetic agents that can counteract immunosuppressive effectors such as tumor-infiltrating myeloid-derived suppressor cells, which can promote the proliferation and the dissemination of residual cancer cells after surgery. Shi et al. focus on the implication of neurotransmitters and beta-adrenergic receptors in neoplastic progression.

Furthermore, the present thematic issue reflects efforts to design novel clinical predictors. Thus, Zheng et al. describe a prognostic model based on the differential expression of RNA binding proteins induced by anesthetics in cervical squamous cell carcinoma. The team of Andresciani et al. suggests the PERIDIAphragmatic surgery score (=PERIDIA-score), which is based on a combination of validated pre-existing scores for specific perioperative medical strategies to improve safety and patient care such as pre-habilitation or physiotherapy.

Two prospective clinical studies deal with the control of surgical stress. Based on results from a randomized controlled trial, Cho et al. hypothesize that dexmedetomidine might sustain anticancer immunity, alleviate surgery-associated inflammatory responses and positively affect long-term therapeutic outcomes. Hu et al. report on pre-habilitation strategies in a single-center, double-blind prospective trial. The authors describe that, compared with both fasting and placebo, the intake of carbohydrates and water before the resection of prostate cancer attenuate the postoperative surge of inflammatory markers such as interleukin 6 (IL-6), IL-8 and tumor necrosis factor alpha (TNFα) in the serum of patients.

Finally, further reviews suggested a role of anesthetic regimens on cancer outcomes. The review of Liu and Wang provides a broad overview on the effects of local, regional or general anesthesia on tumor cells and immune effectors, the risk of recurrence according to the surgical stress and the immunomodulatory properties of various anesthetic agents. Based on preclinical data and retrospective evidence, Ramirez and Cata summarize the consequence of surgery, anesthetic agents (intravenous hypnotics, analgesics, inhalational agents), and the employment of regional *versus* general anesthesia on cancer progression with a particular focus on immune cells present in the tumor microenvironment. Based on preclinical and clinical readouts, Kim et al. also discuss the mechanisms through which surgical stress responses, opioids, and inhalation anesthesia may suppress T cell-mediated immunity and promote distant metastasis. The manuscript of Buddeberg and Seeberger completes this topic by discussing recent data on analgesics such as steroids, alpha-2 agonists or ketamine and by introducing the putative immunologic risks of blood transfusion. In a brief narrative, Montejano and Jevtovic-Todorovic efficiently summarize the potential benefit of intravenous or regional anesthesia contrasting with the risk of relapse increased by volatiles and opioids. **Figure 1**.

**Figure 1:**
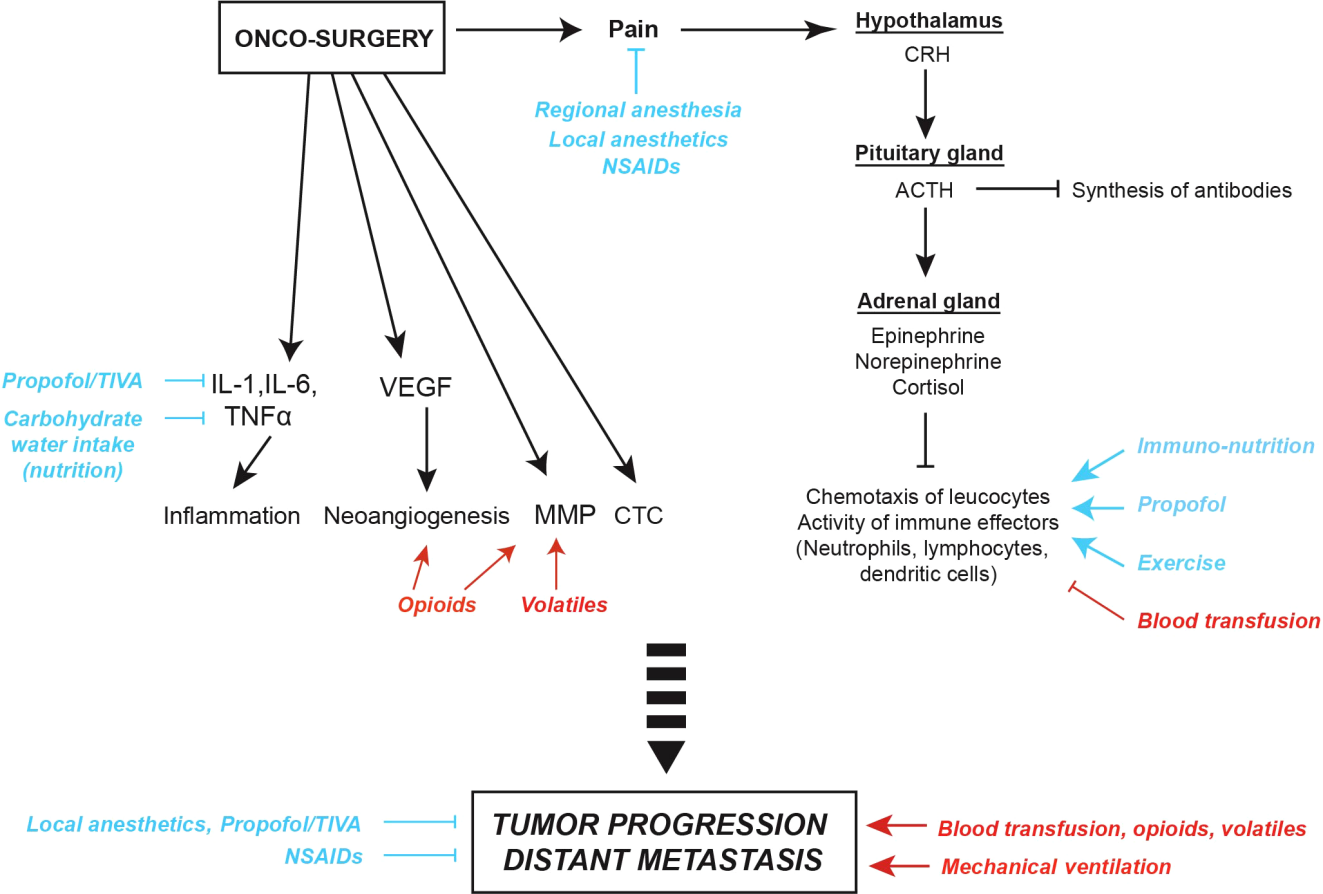
Surgical stress and potential anti- and pro-tumor effects of anesthesia, analgesia and peri-operative factors. Surgical pain can trigger corticotropic signaling, thus favoring the release of endocrine neurotransmitters such as epinephrine, norepinephrine and cortisol. Moreover, metabolic changes induced by surgical stress can impact on the chemotaxis of leucocytes and, as a consequence, induce leukopenia and suppress anticancer immune responses. Oncological procedures can also activate local and systemic inflammatory responses reflected by the release of interleukin-1 (IL-1), IL-6 and tumor necrosis factor alpha (TNFα), trigger the synthesis of vascular endothelial growth factor (VEGF) promoting neoangiogenesis, and increase the release of matrix metalloproteinases (MMP) facilitating the dissemination of circulating tumor cells (CTC). Thus, anesthetic agents and peri-operative factors may mediate either pro- (red) or anti-tumor effects (blue). ACTH, Adreno CorticoTropic Hormone; CRH, Corticotropin-Releasing Hormone; NSAIDs, Non-Steroidal Anti-Inflammatory Drugs; TIVA, Total IntraVenous Anesthesia.

## Conclusion

Onco-anesthesia is a hot topic and has become a research priority. The articles published in this Research Topic summarize recent findings in the field and underline the impact of anesthetic and analgesic procedures as well as that of perioperative care on cancer outcomes. The editors deeply thank all authors, reviewers and co-editors for their fruitful work and thoughtful implication.

## Author contributions

All authors have made a substantial, direct, and intellectual contribution to the work and approved it for publication.
